# Factors Associated with Delayed Diagnosis among Patients with COVID-19 in Okinawa, Japan

**DOI:** 10.3390/ijerph19148634

**Published:** 2022-07-15

**Authors:** Hinako Yamaguchi Oiwake, Daisuke Nonaka, Takehiko Toyosato

**Affiliations:** Graduate School of Health Sciences, University of the Ryukyus, 207 Uehara, Nishihara-cho, Nakagami-gun, Okinawa 903-0215, Japan; nonakad@med.u-ryukyu.ac.jp (D.N.); toyosato@med.u-ryukyu.ac.jp (T.T.)

**Keywords:** COVID-19, delayed diagnosis, onset, testing, age, occupation, route of infection, Okinawa, Japan

## Abstract

The delayed presentation and diagnosis of COVID-19 can contribute to spread of the disease to others but can also cause severe conditions. This study examined factors associated with delayed diagnosis among patients with COVID-19 in Okinawa, Japan. We used the data from 7125 reported cases of people living in Okinawa prefecture with symptom onset between September 2020 and March 2021. The outcome variable was the number of days from symptom onset to diagnosis. The predictor variables included age, sex, occupation, residential area, presumed infection route, and the day of the week. Cox regression analysis was used to compare the outcome between categories for each predictor variable. The median number of days from onset to diagnosis was 3 days, with an interquartile range of 1 to 5 days. Significantly more time from onset to diagnosis was observed in patients in their 60s vs. those in their 20s (hazard ratio: 0.88; 95% confidence interval: 0.81–0.96); hospitality workers were compared to office workers (0.90; 0.83–0.97), patients with unknown infection routes to those with known infection routes (0.77; 0.70–0.84), and those with symptom onset on Sundays/national holidays to those with symptom onset on weekdays (0.90; 0.85–0.96).

## 1. Introduction

In Japan, to prevent the spread of the new coronavirus infection (COVID-19), emergency declarations were issued four times (7 April to 25 May 2020, 8 January to 21 March 2021, 25 April to 20 June 2021, and 12 July to 30 September 2021) as of January 2022, urging the public to restrain from unnecessary outings. In Japan, polymerase chain reaction (PCR) tests or antigen tests are freely available at medical institutions for those with symptoms of suspected coronavirus [1]. If the test result is positive, the patient is, in principle, hospitalized or quarantined at a hotel, prepared by the prefectural government; with the spread of the infection, many people were treated at home due to a shortage of hospital beds. The public health centers conduct an epidemiological survey of those who test positive by calling them. In addition, the public health department recommends testing for close contacts in epidemiological studies [2].

According to similar studies and published data from Okinawa Prefecture, the number of days between the onset of symptoms and diagnosis varies among individuals. Early diagnosis is critical in terms of preventing the spread of infection. It has been reported that novel coronavirus infections may be most infectious at three days before the onset of symptoms to five days after onset [3]. In fact, the Japanese government recommends rapid testing after the onset of symptoms [4]. In addition, minimizing the number of days from onset of symptoms to diagnosis can help prevent serious conditions. In patients with severe conditions, it was reported that symptoms rapidly worsen and may become pneumonia within five to seven days of onset [5].

However, factors associated with a delayed diagnosis have not been adequately clarified. It was reported that, in Hong Kong, living in public rental housing or low-education areas; in Osaka, living outside Osaka City or being infected in early stages of the pandemic are factors associated with a delayed diagnosis; in six prefectures in Japan, an unknown route of infection is associated with delayed diagnosis [6,7,8].

Okinawa Prefecture is the most southerly island of Japan’s 47 prefectures. Although Okinawa is a regional city, it is one of Japan’s most popular tourist destinations, attracting a higher percentage of young people vs. other prefectures [9]. With the spread of infection in urban areas and increased population flow [10], new infections per 100,000 in Okinawa Prefecture has always been higher than in other prefectures [11]. As of 31 July 2021, 24,761 infected people were confirmed in Okinawa, of whom 236 died [12].

The purpose of this study was to determine the factors associated with the number of days between the onset of symptoms and diagnosis depending on demographic and socioeconomic factors, the estimated route of infection, the month of symptom onset, and the day of the week. For our study, we used the data published by Okinawa Prefecture.

## 2. Materials and Methods

### 2.1. Study Design and Data Source

In this observational study, we decided to use secondary data from the Okinawa prefecture government website [12] (https://www.pref.okinawa.jp/site/hoken/kansen/soumu/press/20200214_covid19_pr1.html#youseishaichiran) (accessed on 17 May 2022). Published data included the following informative items: date of symptom onset, date of diagnosis, sex, age, city of residence or jurisdictional health center, occupation, and presumed route of infection.

### 2.2. Patients

We included 10,214 laboratory-confirmed patients with COVID-19 reported by the Okinawa prefectural government, with illness onset between 1 September 2020 and 31 March 2021. Other periods were not included in our study, as demand for testing may have exceeded supply due to the spread of infection, and testing may not have been available with the desired timing [9,10]. We excluded patients with “missing data on either the date of onset of symptoms or diagnosis” (*n* = 3057), “sex, age, or occupation unknown” (*n* = 25), and “others” (*n* = 7). A total of 7125 patients were included.

### 2.3. Outcome Variable

The outcome variable was the number of days from onset of symptoms to diagnosis. It was calculated by subtracting the number of days from diagnosis to symptom onset. In other words, a patient with 0 days between the onset of symptoms and diagnosis was diagnosed with the novel coronavirus on the day of onset.

### 2.4. Predictor Variables

There were seven predictor variables. Residential areas were classified into six groups according to the jurisdictional public health center. Table A1 shows the municipalities under the jurisdiction of the public health center.

Occupations were categorized into eight groups based on the type of work, employment status, and ease of taking time off. “Office workers” included military base workers, association staff members, civil servants, administrative staff, real estate agents, educators, company executives, and those in sales positions. “Hospitality workers” included restaurant, service, and hospitality industry workers and salespersons. “Medical professionals and care workers” included facility staff. “Other” groups included part-time workers, those self-employed, childcare workers, transport workers, farmers, livestock producers, fishermen, sailors, assemblymen, and cleaners. Detailed variables are shown in Table A2.

The presumptive routes of infection were categorized by three groups: “contact with confirmed cases,” “unknown infection route,” and “under investigation.” “Contact with confirmed cases” included “contact in/outside the prefecture,” as well as “infection in the workplace.” “Unknown infection route” included “travel outside the prefecture.”

The day of the week (weekday, Saturday, or Sunday/holiday) and the month of symptom onset were also used as predictor variables to track access to the fever outpatient clinic.

### 2.5. Statistical Analyses

Descriptive statistics were used to summarize data using medians, 95% confidence intervals, and interquartile ranges of the days from onset of symptoms to diagnosis for each group, as well as the mean for ease of understanding. As a bivariate analysis, we constructed Kaplan–Meier survival plots for each outcome, along with each predictor variable and a log-rank test to compare groups. As a multivariate analysis, we used Cox proportional-hazards models to calculate hazard ratios (HRs) and 95% confidence intervals (CIs). Two models were examined: Model 1, where all variables were included, and Model 2, where only predictor variables, significantly different according to the log-rank test, were included. Significance was defined as *p* < 0.05 (two-sided). These analyses relied on IBM SPSS Statistics for Windows, Version 25.0 (IBM Corp., Armonk, NY, USA).

## 3. Results

### 3.1. Characteristics of Patients

Table 1 shows the basic patient characteristics. Male cases accounted for 56.3% of the population. The most common age group was individuals in their 20s (22.2%), followed by those in their 40s (15.4%). The most common occupation was office worker (26.5%), followed by unemployed (16.1%), and hospitality worker (13.9%). The most common presumptive route of infection was contact with confirmed cases (77.7%). The month and day of onset of symptoms with most cases turned out to be January (28.6%) and weekdays (72.2%). Moreover, 20.7% of patients had unreported occupations, and 15.3% had an unknown route of infection.

The overall median and mean days from the onset of symptoms to diagnosis was 3 days (IQR: 1–5; 95% CI: 2.94–3.06). Table 2 shows the time from the onset of symptoms to diagnosis, according to the characteristics of cases. The shortest number of median days (1 day) was seen in the oldest age group (90 or over), whereas the highest number of median days (4 days) was seen in patients with an unknown route of infection.

### 3.2. Bivariate Analysis

**Figure 1:**
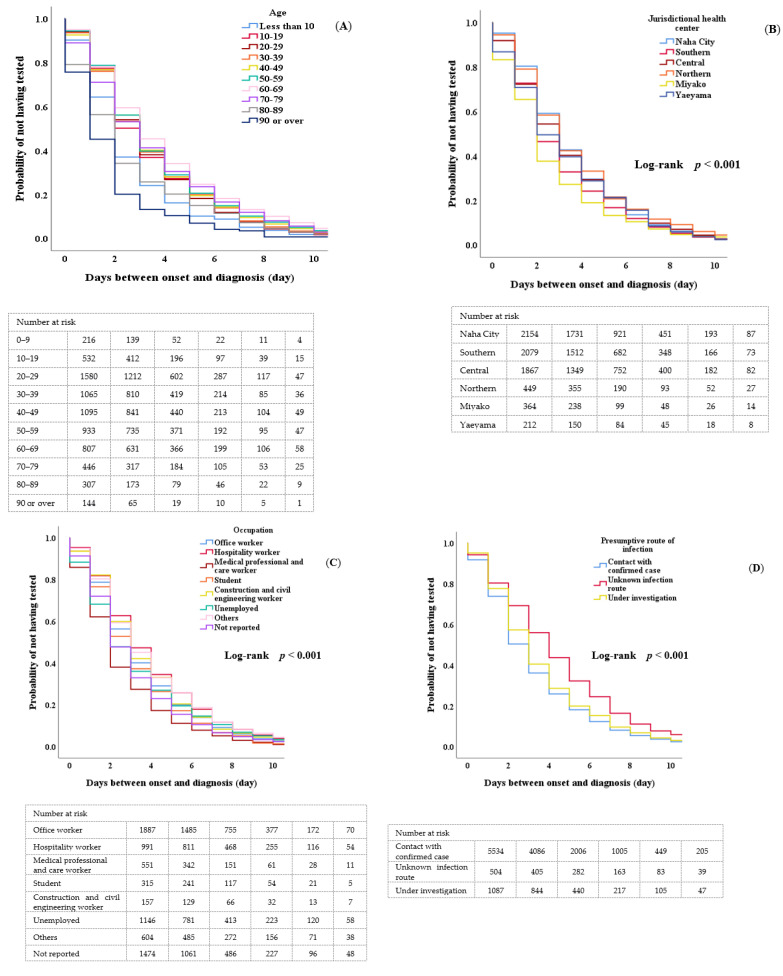

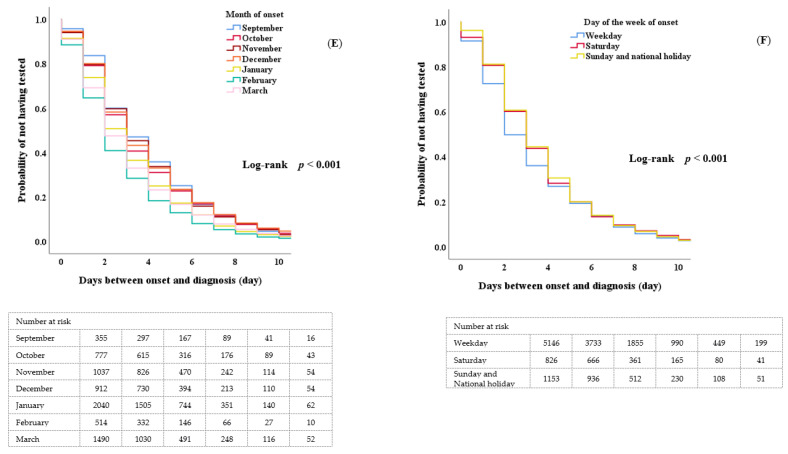
Survival Analyses (**A**) Age, (**B**) Jurisdictional health center, (**C**) Occupation, (**D**) Presumptive route of infection, (**E**) Month of onset, (**F**) Day of the week of onset.

Figure 1 shows the Kaplan–Meier curve of the days from the onset of symptoms to diagnosis by age, jurisdictional health center, occupation, presumptive route of infection, month of onset, and day of the week of onset for COVID-19 patients in Okinawa. The log-rank test showed significant differences between two survival rates for age, jurisdictional health center, occupation, estimated route of infection, month of onset, and day of onset (*p* < 0.001, respectively). There were no significant differences by sex (*p* = 0.83).

### 3.3. Multivariate Analysis

Table 3 shows hazard ratios (HRs) and 95% confidence intervals (CIs) for the multivariate Cox proportional-hazards model for the days from the onset of symptoms to diagnosis. In Model 1, the number of days from the onset of symptoms to diagnosis was significantly longer for people in their 60s (HR: 0.88; 95% CI: 0.81–0.96) and shorter for those in their 80s (HR: 1.32; 95% CI: 1.15–1.51) and 90s or older (HR: 1.93; 95% CI: 1.60–2.32), vs. those in their 20s. There were significant differences in hazard ratios for jurisdictional health centers. In terms of occupation compared to office workers, they were significantly shorter in “medical professionals and care workers” (HR: 1.40; 95% CI: 1.27–1.54) or “not reported” (HR: 1.14; 95% CI: 1.05–1.23) and longer in “hospitality workers” (HR: 0.90; 95% CI: 0.83–0.97) and “others” (HR: 0.89; 95% CI: 0.81–0.98). For presumed routes of infection, the days from the onset of symptoms to diagnosis were significantly longer for “unknown infection route” (HR: 0.77; 95% CI: 0.70–0.84) and “under investigation” (HR: 0.83; 95% CI: 0.77–0.90) vs. those in “contact with confirmed cases.” For the month of onset, the days from the onset to diagnosis were significantly shorter in “January” (HR: 1.16; 95% CI: 1.04–1.31), “February” (HR: 1.41; 95% CI: 1.22–1.62), and “March” (HR: 1.28; 95% CI: 1.13–1.45) than “September.” By the day of the week for onset, the days from the symptom onset to diagnosis were significantly longer for “Saturdays” (HR: 0.92; 95% CI: 0.85–0.99) and “Sunday and national holidays” (HR: 0.90; 95% CI: 0.85–0.96) than “Weekdays.” Model 2 showed significant differences for the same factors as Model 1.

## 4. Discussion

In this study, using laboratory-confirmed cases in Okinawa Prefecture from September 2020 through March 2021, we attempted to find an association between the number of days from the onset of symptoms to the diagnosis of COVID-19 and sociodemographic factors, presumed route of infection, and timing of disease onset. We saw that significantly more time from the onset of symptoms to diagnosis was observed in patients in their 60s (vs. those in their 20s), hospitality workers (vs. office workers), patients with an unknown route of infection (vs. those with a known route of infection), and patients with symptom onset on a weekend/national holiday (vs. those with onset on a weekday).

The diagnosis was delayed for those with unknown routes of infection and those under investigation for route of infection. The results are supported by a study in six prefectures in Japan [13]. The delayed diagnoses in these two groups indicate that, even if symptoms of COVID-19 are present, a lack of apparent recognition of contact will delay the time to diagnosis. Since new coronavirus infections present cold-like symptoms and vary among individuals, people may not suspect COVID-19 without contact history. Specific symptoms of COVID-19 are shortness of breath and taste and smell disorders that trigger testing. It has been reported that 30% of infected people have no fever, cough, or shortness of breath [14], and that the prevalence of taste and smell disorders was 44% and 52%, respectively [15]. Hence, it is necessary to further publicize the importance of testing with the appearance of any symptoms, even if there is no contact history. Close contacts can be tested promptly and at the appropriate time for minor symptoms, with guidance from the public health center for administrative testing; those under investigation for route of infection included 15.3% of participants. Although published data may not be updated, public health centers may have been understaffed at the time of outbreak or inadequately investigated [16]. We must develop a system with appropriate epidemiological investigations, allowing patients to input their own data.

Furthermore, we found that diagnosis was delayed for those with the onset of symptoms on weekends and holidays. Many medical clinics are closed on weekends, which may have been a factor. If symptoms are mild, many people may not go to the emergency department, where tests are available, and may test later in the week.

In the present study, diagnosis was delayed in people in their 60s vs. in their 20s. This finding is consistent with a study in Shaanxi Province, China, showing that people over 60 were tested later than younger people [17]. It was reported that “in the elderly, fever is absent or blunted in 20–30% of infections,” the infection may not be recognized” [18], and diagnosis may be delayed. In addition, low health literacy in older adults [19], social isolation [20,21] due to retirement, and children’s independence could have affected the delay in diagnosis for those in their 60s. Older adults may accept some symptoms, as they do not want to bother others about being infected [22]. Improving access to testing for those in their 60s, at high risk of serious illness, could also contribute to reducing healthcare costs, as well as preventing the spread of infection.

In contrast, we found that people in their 80s and 90s or older were diagnosed earlier. Several facility clusters were reported in Okinawa [23], so we assume that some elderly people were in care facilities or hospitalized. However, information on the type of residence and family support was unavailable for data in this study. If one resides in an elderly care facility, when a cluster occurs, other older adults are promptly screened [24,25,26]. This system allows for the early detection of infection even if older adults are unaware of their symptoms or have difficulty reporting them. Encouragement to report symptoms promotes health-seeking behavior [27]. As such, older adults are at higher risk of serious illness, with constant attention being paid to their physical condition by family members and medical professionals, which is associated with faster diagnosis. This study found that the time to diagnosis differed by age, even in the elderly over 60 years, classified in the same group in other studies. This suggests that different physical and social characteristics must be analyzed separately.

Our findings suggest that people living in the southern (and Miyako Health Center) area were diagnosed earlier than those in Naha City. There is no clear explanation for this. Findings support the Osaka study, showing that people living outside government-designated cities had faster diagnoses [7]. The southern region and the Miyako Island region are a mix of rural and urban areas, some of which are also islands, meaning geographical access to medical institutions is not easy. Public health centers have an essential role in controlling infectious diseases in Japan [2]. The significant differences in health centers may be related to their effort to conduct testing of contacts, referrals to medical institutions, and differences among local laboratories. Identifying local factors ensures the continued availability of resources for COVID-19 measures [28]; testing must be recommended in areas where diagnoses are delayed to strengthen the system, to staff public health centers, and to analyze factors in which diagnosis is early.

Considering occupation, the time to diagnosis was delayed for hospitality workers. In the restaurant industry, non-regular employees are 80% of the workforce in Japan [29]. Many may be discouraged from testing due to economic instability and the lack of paid leave. As such, those in other occupations, of whom 75.3% were part-timers and self-employed, were considered as diagnosed late. Paid sick leave is necessary for infectious diseases [30,31]; many developed countries, including Australia, New Zealand, The Netherlands, Switzerland, and the UK, have universal access to paid sick leave, while Canada, Japan, and the USA do not have universal access to paid leave, even for full-time employees [32]. If non-regular employees test positive for COVID-19, they are not paid for sick leave; they are more likely to be reluctant to test vs. regular employees. As the percent of non-regular employees increases in Japan [33], measures that consider this type of employment are crucial. It is necessary to compensate for income due to sick leave for non-regular employees and to promote use of paid leave for regular employees.

In terms of month of onset, patients with the onset of symptoms between January and March 2021 were diagnosed sooner than those with symptom onset in September 2020. There are three potential reasons for this: the expansion of testing, changing public awareness, and the influx of variant strains [9]. In January, Okinawa Prefecture began providing regular PCR testing to employees of care service facilities requesting it [34]. From February 2021, applicants and travelers could be tested at PCR centers in Naha City and Naha airports [35,36]. With the expansion of PCR testing, it is now available outside medical institutions for faster diagnosis; the number of infected people and the declaration of a state of emergency (1 August to 5 September 2020 and 19 January to 28 February 2021) could have changed testing behavior. Okinawa Prefecture confirmed 364 positive cases in September 2020, with 2154 in January 2021, 635 in February, and 1365 in March [12] and declared a state of emergency from 1 to 5 September 2020 and 19 January to 28 February 2021. We assume that media information regarding the influx of variant strains improved awareness of how people could be infected with COVID-19. These results coincide with previous studies in Osaka, i.e., that the time to testing and diagnosis became shorter each month [7,37].

We acknowledge three limitations in the study. First, we could not examine some relevant factors not included in the present data. Symptom severity at the time of testing, the type of test used, underlying diseases, fear of stigma, and transportation may be associated with testing behavior [27]. Further research is needed to confirm factors linked to time and diagnosis, which must focus on variable behavioral factors.

Second, this study failed to incorporate patients’ vaccination status, which affects the onset of symptoms and testing. The impact of this limitation is minimal, as vaccinations were limited to healthcare professionals during the study period.

Third, public health centers conduct active epidemiological surveys as part of their cluster control measures, detecting contact cases more efficiently. Symptom onset date in published data was stated in the occurrence report by physicians who performed the testing, but there is a possibility of bias due to method of questioning and recall bias.

Our findings suggest a need to strengthen strategies to target populations with delayed diagnoses. Socioeconomic disparities in health, including infectious diseases [38], are associated with public health strategies, which must consider them. It is critical to establish easily accessible laboratories, regardless of the day of the week or area of residence; this strengthens the strategies of public health centers and creates a work environment in which it is easy to go for testing.

## 5. Conclusions

This study shows that the number of days from the onset of symptoms to diagnosis with COVID-19 was significantly associated with age, occupation, residential area, presumed infection route, and onset day; a greater number of days was observed for patients in their 60s (vs. those in their 20s), patients who were hospitality workers (vs. those who were office workers), patients with unknown infection routes (vs. those with known infection routes), and patients with symptom onset on weekends/national holidays (vs. those with symptom onset on weekdays). These results suggest that Japanese national and local governments can improve delayed presentation and diagnosis for COVID-19 with attention to the attitudes of those in their 60s, improved access to diagnosis for hospitality workers and non-regular employees, as well as improving access to diagnosis on weekends/national holidays.

## Figures and Tables

**Table 1:** Characteristics of persons with confirmed COVID-19 infection in Okinawa between 1 September 2020 and 31 March 2021 (*n* = 7125).

Variables	*n*	(%)
Sex		
Male	4012	(56.3)
Female	3113	(43.7)
Age		
Less than 10	216	(3.0)
10–19	532	(7.5)
20–29	1580	(22.2)
30–39	1065	(14.9)
40–49	1095	(15.4)
50–59	933	(13.1)
60–69	807	(11.3)
70–79	446	(6.3)
80–89	307	(4.3)
90 or over	144	(2.0)
Jurisdictional health center		
Naha City	2154	(30.2)
Southern	2079	(29.2)
Central	1867	(26.2)
Northern	449	(6.3)
Miyako	364	(5.1)
Yaeyama	212	(3.0)
Occupation		
Office worker	1887	(26.5)
Hospitality worker (restaurant and service)	991	(13.9)
Medical professional and care worker	551	(7.7)
Student	315	(4.4)
Construction and civil engineering worker	157	(2.2)
Unemployed	1146	(16.1)
Others	604	(8.5)
Not reported	1474	(20.7)
Presumptive route of infection		
Contact with confirmed case	5534	(77.7)
Unknown infection route	504	(7.1)
Under investigation	1087	(15.3)
Month of onset		
September	355	(5.0)
October	777	(10.9)
November	1037	(14.6)
December	912	(12.8)
January	2040	(28.6)
February	514	(7.2)
March	1490	(20.9)
Day of the week of onset		
Weekday	5146	(72.2)
Saturday	826	(11.6)
Sunday and national holiday	1153	(16.2)

**Table 2:** The number of days from symptom onset to diagnosis for each group.

Variables	Survival Time			
	Median	IQR *	Mean	95% CI ^†^
Sex				
Male	3	(1–5)	3.48	3.39–3.57
Female	3	(2–5)	3.46	3.36–3.56
Age				
Less than 10	2	(1–3)	2.76	2.37–3.15
10–19	3	(2–5)	3.32	3.11–3.53
20–29	3	(2–5)	3.43	3.29–3.56
30–39	3	(2–5)	3.49	3.32–3.66
40–49	3	(2–5)	3.56	3.39–3.73
50–59	3	(2–5)	3.66	3.47–3.85
60–69	3	(2–5)	4.03	3.80–4.25
70–79	3	(1–5)	3.60	3.31–3.89
80–89	2	(1–4)	2.67	2.34–3.00
90 or over	1	(1–2)	1.83	1.49–2.18
Jurisdictional health center				
Naha City	3	(2–5)	3.69	3.58–3.81
Southern	2	(1–4)	3.22	3.10–3.34
Central	3	(1–5)	3.55	3.42–3.69
Northern	3	(2–5)	3.84	3.55–4.12
Miyako	2	(1–4)	2.84	2.53–3.15
Yaeyama	2	(1–5)	3.34	2.97–3.72
Occupation				
Office worker	3	(2–5)	3.61	3.48–3.73
Hospitality worker (restaurant and service)	3	(2–6)	4.00	3.82–4.18
Medical professional and care worker	2	(1–4)	2.61	2.42–2.81
Student	3	(2–5)	3.30	3.03–3.57
Construction and civil engineering worker	3	(2–5)	3.70	3.28–4.12
Unemployed	2	(1–5)	3.36	3.18–3.55
Others	3	(2–6)	4.01	3.75–4.27
Not reported	2	(1–4)	3.15	3.01–3.29
Others				
Presumptive route of infection				
Contact with confirmed case	3	(1–5)	3.34	3.27–3.41
Unknown infection route	4	(2–6)	4.55	4.26–4.84
Under investigation	3	(2–5)	3.66	3.48–3.83
Month of onset				
September	3	(2–6)	3.95	3.65–4.24
October	3	(2–5)	3.77	3.56–3.98
November	3	(2–5)	3.85	3.67–4.03
December	3	(2–5)	3.90	3.97–4.10
January	3	(1–4)	3.28	3.17–3.40
February	2	(1–4)	2.78	2.56–3.00
March	2	(1–4)	3.18	3.04–3.33
Day of the week of onset				
Weekday	2	(1–5)	3.37	3.29–3.45
Saturday	3	(2–5)	3.70	3.51–3.90
Sunday and national holiday	3	(2–5)	3.78	3.62–3.95

* Interquartile range, ^†^ 95% confidence interval.

**Table 3:** Cox regression analysis of days from symptom onset to diagnosis (Model 1).

Variables	Hazard Ratio	95% CI ^†^	*p*-Value
Sex			
Male	Ref		
Female	0.96	0.91–1.01	0.077
Age			
Less than 10	1.07	0.92–1.25	0.400
10–19	0.95	0.85–1.06	0.359
20–29	Ref		
30–39	1.00	0.92–1.08	0.959
40–49	1.00	0.92–1.08	0.962
50–59	0.96	0.88–1.05	0.343
60–69	0.88	0.81–0.96	0.005
70–79	1.01	0.90–1.14	0.826
80–89	1.32	1.15–1.51	<0.001
90 or over	1.93	1.60–2.32	<0.001
Jurisdictional health center			
Naha City	Ref		
Southern	1.12	1.06–1.19	<0.001
Central	1.02	0.96–1.09	0.510
Northern	0.97	0.87–1.08	0.550
Miyako	1.29	1.15–1.44	<0.001
Yaeyama	1.08	0.93–1.25	0.303
Occupation			
Office worker	Ref		
Hospitality worker (restaurant and service)	0.90	0.83–0.97	0.009
Medical professional and care worker	1.40	1.27–1.54	<0.001
Student	1.01	0.89–1.15	0.901
Construction and civil engineering worker	0.94	0.80–1.11	0.461
Unemployed	0.97	0.89–1.06	0.520
Others	0.89	0.81–0.98	0.014
Not reported	1.14	1.05–1.23	<0.001
Presumptive route of infection			
Contact with confirmed case	Ref		
Unknown infection route	0.77	0.70–0.84	<0.001
Under investigation	0.83	0.77–0.90	<0.001
Month of onset			
September	Ref		
October	1.00	0.88–1.14	0.997
November	1.04	0.92–1.18	0.505
December	1.01	0.90–1.15	0.829
January	1.16	1.04–1.31	0.010
February	1.41	1.22–1.62	<0.001
Day of the week of onset			
Weekday	Ref		
Saturday	0.92	0.85–0.99	0.024
Sunday and national holiday	0.90	0.85–0.96	<0.001

^†^ 95% confidence interval.

## Data Availability

The data are available at the Supplementary Materials.

## Supplementary Materials

The following supporting information can be downloaded at: https://www.mdpi.com/article/10.3390/ijerph19148634/s1, Spreadsheet S1: Dataset.

## Appendix A

**Table A1:** Municipalities under the jurisdiction of health centers.

Health Center	Jurisdictional Municipality		
	City	Town	Village
Naha City	Naha		
Southern	Itoman Urasoe Tomigusuku Nanjo	Nishihara Haebaru Yaese Kumejima *	Tokashiki * Zamami * Aguni * Tonaki * Minamidaito * Kitadaito *
Central	Ginowan Okinawa Uruma	Kin Kadena Chatan	Onna Ginoza Yomitan Kitanakagusuku Nakagusuku
Northern	Nago	Motobu	Kunigami Ogimi Higashi Nakijin Ie * Iheya * Izena *
Miyako	Miyakojima *		Tarama *
Yaeyama	Ishigaki *	Taketomi * Yonaguni *	

* The municipality itself is separated from the main island, and the island is not connected by a bridge.

**Table A2:** Detailed description of occupation variable.

Group	Occupations
Office worker	Office worker/military base worker/association staff member civil servant/administrative staff/real estate agent/educator company executive/sales position
Hospitality worker (restaurant and service)	Restaurant business/service worker/hospitality industry salesperson
Medical professional and care worker	Medical professional/care worker/facility staff
Student	Student
Construction and civil engineering worker	Construction worker/civil construction engineering worker
Unemployed	Unemployed
Others	Part-time worker/self-employed person/childcare worker transport worker/farmer/livestock producer/assemblyman fisherman/sailor/cleaner
Not reported	Not reported
